# Tinnitus- related distress: evidence from fMRI of an emotional stroop task

**DOI:** 10.1186/s12901-016-0029-1

**Published:** 2016-08-05

**Authors:** Dennis Golm, Carsten Schmidt-Samoa, Peter Dechent, Birgit Kröner-Herwig

**Affiliations:** 1 Department of Clinical Psychology and Psychotherapy, Georg-August-University, Georg-Elias-Mueller-Institute of Psychology, Gosslerstrasse 14, 37073 Goettingen, Germany; 2 Georg-August-University, UMG, MR-Research in Neurology and Psychiatry, Robert-Koch-Str. 40, 37075 Goettingen, Germany; 3 University of Southampton, Academic Unit of Psychology, Developmental Brain Behaviour Laboratory, Highfield Campus, Building 44, SO17 1 BJ Southampton, UK

## Abstract

**Background:**

Chronic tinnitus affects 5 % of the population, 17 % suffer under the condition. This distress seems mainly to be dependent on negative cognitive-emotional evaluation of the tinnitus and selective attention to the tinnitus. A well-established paradigm to examine selective attention and emotional processing is the Emotional Stroop Task (EST). Recent models of tinnitus distress propose limbic, frontal and parietal regions to be more active in highly distressed tinnitus patients. Only a few studies have compared high and low distressed tinnitus patients. Thus, this study aimed to explore neural correlates of tinnitus-related distress.

**Methods:**

Highly distressed tinnitus patients (HDT, *n* = 16), low distressed tinnitus patients (LDT, *n* = 16) and healthy controls (HC, *n* = 16) underwent functional magnetic resonance imaging (fMRI) during an EST, that used tinnitus-related words and neutral words as stimuli. A random effects analysis of the fMRI data was conducted on the basis of the general linear model. Furthermore correlational analyses between the blood oxygen level dependent response and tinnitus distress, loudness, depression, anxiety, vocabulary and hypersensitivity to sound were performed.

**Results:**

Contradictory to the hypothesis, highly distressed patients showed no Stroop effect in their reaction times. As hypothesized HDT and LDT differed in the activation of the right insula and the orbitofrontal cortex. There were no hypothesized differences between HDT and HC. Activation of the orbitofrontal cortex and the right insula were found to correlate with tinnitus distress.

**Conclusions:**

The results are partially supported by earlier resting-state studies and corroborate the role of the insula and the orbitofrontal cortex in tinnitus distress.

## Background

Tinnitus refers to the perception of sounds with no external origin [1]. Chronic tinnitus affects approximately 5 % of the population [2, 3]. While most individuals habituate to this phantom noise, 17 % of the individuals with chronic tinnitus are however severely distressed by the condition [4]. This distress is not predicted by psychoacoustic qualities of the tinnitus [5, 6], but is rather due to a negative initial cognitive- emotional evaluation of the tinnitus sound [7].

Dysfunctional beliefs about tinnitus, attention focus on the tinnitus, dysfunctional coping and avoidance behavior are considered to instigate and maintain tinnitus- related distress [8, 9]. Indeed, it has been shown that subjects with unilateral tinnitus pay more attention to the tinnitus ear [10]. Furthermore, this attention focus on tinnitus seems to increase tinnitus- related distress [11]. Concluding from those studies, people with tinnitus focus their attention on the phantom noise and this in turn elevates the tinnitus- related distress. On the other hand, it has been shown that attention to tinnitus is influenced by the amount of tinnitus annoyance [12]. Thus, attentional bias to tinnitus seems to be influenced by the amount of tinnitus- related distress. Additionally, Andersson and Westin [13] suggested attention to tinnitus as a mediator for tinnitus- related distress, provided that tinnitus is appraised negatively. This view has been corroborated by a study of Cima and colleagues [14], who found an association between catastrophizing and increased attention towards tinnitus in a sample of 61 tinnitus patients and by Andersson and collaborators [15] who could show that attention to tinnitus increased the amount of tinnitus- related thoughts compared to thought- suppression. Thus, there seems to be an association between attention focus to tinnitus and tinnitus- related negative information. Support for this view comes from a study that found a facilitation effect towards tinnitus- related words in comparison to neutral words measured by the Emotional Stroop Task (EST) in a group of tinnitus patients, but not in a control group [16].

The EST is a well- established paradigm to examine emotional processing [17–19] and attentional bias [20]. It has been frequently used in the field of emotional disorders [20] and also in chronic pain [21] which shares common features with tinnitus [22–24]. Emotionally salient words should draw attention from the task (color- naming of the words), thus resulting in longer reaction times [25]. Generally, studies on the EST find an interference- effect for concern- related words. Andersson and colleagues [16] on the other hand found a facilitation effect for tinnitus- related words within a group of tinnitus patients (*n* = 104), but not within a healthy control group (*n* = 21). However, this study had some methodological issues, since the groups were not compared with each other and varied greatly in sample size. Another study on tinnitus patients that used the EST did not find any interference or facilitation effect for tinnitus- related words [26]. Thus, there seems to be no clear evidence of an Emotional Stroop effect in tinnitus patients. However, none of these studies controlled for the level of tinnitus- related distress as a potential moderator of effects. Therefore, we expect an Emotional Stroop effect to only occur in highly distressed tinnitus patients. No study known to the authors has ever examined an Emotional Stroop effect in highly compared to low distressed tinnitus patients.

Additionally, the emotional processing of tinnitus-related words should heighten the tinnitus annoyance, resulting in the activation of distress-related brain regions. However, little is known about the neural correlates of tinnitus related distress. According to the Global Brain Model [27], damage to the hearing system reduces the sensory input, decreases inhibitory mechanisms in the central auditory system and finally leads to an enhanced excitability of the auditory cortices. This activity in the auditory cortices is supposed to be modulated by a network consisting of frontal, parietal and cingulate regions. The model proposes that this fronto- parietal- cingulate network is more active in highly distressed tinnitus patients. The dorsolateral prefrontal cortex (DLPFC), the orbitofrontal cortex (OFC), anterior cingulate (ACC) and the precuneus/posterior cingulate (PCC) are considered as key structures in that network. A resting- state electroencephalography (EEG) study [28] identified a component, that differed between high and low distressed tinnitus patients (14–18 Hz, 22–26 Hz) that consisted of the medial frontal gyrus, middle frontal gyrus, inferior frontal gyrus, rectal gyrus, ACC, parahippocampal gyrus and the insula. Another resting- state EEG study that compared high and low distressed tinnitus patients [29] identified four regions that contributed significantly to tinnitus annoyance; the subcallosal ACC, the parahippocampal area, the PCC and the DLPFC. Further support for this model comes from resting- state fMRI- studies. In a mixed sample of bothered and non- bothered tinnitus patients according to the Tinnitus Questionnaire (TQ) [30], tinnitus patients showed higher functional connectivity within an auditory resting-state network in comparison to healthy controls bilaterally in the parahippocampal gyrus, the inferior frontal gyrus, right prefrontal cortex, right inferior parietal lobe and postcentral gyrus [31]. A resting- state fMRI- analysis on bothered tinnitus patients showed greater functional connectivity as compared to HC between the right anterior insula and left inferior frontal gyrus which correlated positively with activity in the auditory cortex [32]. No differences in functional connectivity could be found in a comparison of non- bothered tinnitus patients and healthy controls [33]. Thus, these studies confirmed the role of frontal and limbic structures in tinnitus distress and to some extent in parietal areas. A resting state Magnetoencephalography study found a correlation between the strength of inflow to the temporal cortices and tinnitus annoyance. The temporal cortices received that input from the prefrontal cortex (PFC), cuneus, precuneus and PCC [34]. Hence, corroborating a role of the precuneus in tinnitus annoyance.

Recently, it has been suggested that several overlapping brain networks contribute to the perception of tinnitus; the somatosensory cortex, the auditory cortex, a perception network, a salience network, a distress network and memory areas [35]. Networks of interest for the study of selective attention and distress are the perception network, salience network, distress network and memory areas. Subgenual ACC, dorsal ACC, PCC, parietal cortex, the precuneus and the frontal cortex form the perception network. Activity within these areas is required to perceive a phantom percept consciously. The salience network, consisting of the dorsal PCC and anterior insula reflects the behavioral significance of the percept. The distress network should include the ACC, anterior insula and amygdala. According to the model memory areas; the parahippocampal area, hippocampus and amygdala, should be associated with awareness to the salient perception and play a role in the reinforcement of annoyance [35, 36].

Based on the available empirical evidence regarding tinnitus distress and taking into account the suggestions of the Global Brain Model and the Working Model of Phantom Percepts we hypothesize highly distressed tinnitus patients (HDT) to react slower (interference- effect) to tinnitus-related words as compared to neutral words in an EST and in comparison to low distressed tinnitus patients (LDT) and healthy controls (HC). Additionally, we expect HDT to rate tinnitus- related words as being more negative and arousing in comparison to neutral words and in comparison to LDT and HC. On a neural level we expect HDT to show a higher activity, as measured by blood oxygen level dependent (BOLD) fMRI, in the precuneus, limbic areas and frontal areas in comparison to LDT and HC, especially the parahippocampus, dorsal and subgenual ACC (including anterior and posterior midcingulate cortex), PCC, insula, DLPFC (Brodman Area (BA) 9, 46) and OFC (including inferior frontal gyrus, BA 10, 11, 47).

## Methods

### Sample

**Fig. 1:**
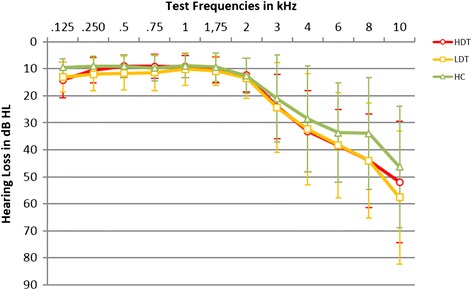
Hearing loss in dB HL. dB= decibel, HC= healthy controls, HDT= highly distressed tinnitus patients, HL= hearing level, LDT= low distressed tinnitus patients, kHz= Kilohertz

Participants were recruited for participation in the study via regional newspapers, the homepage of the German Tinnitus League, flyers and word of mouth. Inclusion criteria were a chronic tinnitus, defined as a constant noise in the ear(s) or the head for at least one year and German as the first language. Exclusion criteria were age above 70, a current major depressive syndrom, hyperacusis, current treatment with psychotropic drugs, days without tinnitus perception, tinnitus perception only in total silence, residual inhibition > one minute, any counter indications to MR- methodology (e.g. pacemaker) and an actual hearing loss. According to the Guidelines on Non- Physician Care and Medical Aids (Heil- und Hilfsmittelrichtlinien) hearing loss was defined as a loss ≥ 30 dB HL at 2 kHz or in two other frequencies between 0.5 kHz and 3 kHz on the better hearing ear [37]. Participants were allocated to the HDT- group if they achieved a score above 30 (moderate annoyance) in the German version of the TQ [30, 38]. The final sample consisted of 48 participants; 16 HDT, 16 LDT and 16 HC. The groups were matched by age and sex. As expected, HDT had a higher level of tinnitus distress. HDT had higher anxiety and depression scores as measured by the German version of the Hospital Anxiety and Depression Scale (HADS) [39, 40] and higher hypersensitivity to sound scores as measured by a Questionnaire on Hypersensitivity to sound (GÜF) [41] than LDT and HC. In comparison to LDT, HDT had a lower vocabulary test score in a subtest of the Hamburg Wechsler Intelligence Test [42]. The three groups did not differ with regard to age, sex, tinnitus loudness and hearing loss (see Table 1 and Fig. 1 for details) (Please see the assessment section for details about the instruments).

**Table 1:** Description of the groups and characterizing variables

	HDT		LDT		HC		HDT vs. LDT	HDT vs. HC	LDT vs. HC
	(*n*=16; 13♂)		(*n*=16; 13♂)		(*n*=16; 13♂)		df=30	df=30	df= 30
	Mean	SD	Mean	SD	Mean	SD	*t (p)*	*t (p)*	*t (p)*
Age	53.38	12.33	52.88	12.14	52.38	9.42	0.12 (0.9088)	0.26 (0.7984)	0.13 (0.8973)
HADS A	8.31	3.42	4.06	3.07	2.75	2.29	3.70 (0.0009)	5.40 (0.0000)	1.37 (0.1805)
HADS D	6.75	3.44	3.38	3.72	2.56	2.58	2.67 (0.0123)	3.90 (0.0005)	0.72 (0.4786)
VT	20.0	5.37	24.38	4.11	21.94	4.20	- 2.59 (0.0147)	- 1.14 (0.2646)	1.66 (0.1077)
GÜF	13.19	8.16	6.06	5.63	2.56	2.22	2.88 (0.0074)	5.03 (0.0000)	2.31 (0.0277)
Hearing Loss	22.23	6.77	23.28	10.41	19.31	9.09	- 0.34 (0.7365)	1.03 (0.3120)	−0.34 (0.7365)
TQ^a^	40.0	6.69	15.0	6.28			10.89 (0.0000)		
Loudness	39.75	20.99	49.94	20.77			- 1.38 (0.1778)		

♂ male, *df* degrees of freedom, *GÜF* Geräuschüberempfindlichkeitsfragebogen (Questionnaire on Hypersensitivity to Sound), *HADS* Hospital Anxiety (A) and Depression (D) Scale, *HC* healthy controls, *HDT* highly distressed tinnitus patients, *LDT* low distressed tinnitus patients, *Loudness* maximum (in case of bilateral tinnitus) loudness of the tinnitus in dB HL as measured via matching of the tinnitus to a similar sound, *t* t- value, *TQ* Tinnitus Questionnaire, *VT* vocabulary test

All t-tests were two-sided

^a^Due to missing data on the day of the MRI- scan, the missing TQ- score of 4 participants (1 HDT, 3 LDT) was replaced with the TQ- score from the TQ, that had been filled in after the telephone screening

### Experimental Design

The Emotional Stroop Task comprised of two conditions; tinnitus- related words (TW) and neutral words (NW). The stimuli were presented in a block- design with six blocks per stimulus category. Within one block, each word was presented for 1750 ms in one of four colors (red, blue, green, yellow), followed by a fixation cross (250 ms). The words were presented in a randomized order and each word was presented twice per block. Thus, the length of each block was 24 s. Neutral blocks alternated with blocks of TW. Before and after each block a fixation cross was presented for 24 s. Participants were instructed to identify the color of each word by pressing a button on a four- button- response- pad by using the index- and middle- finger of each hand. Inside the MRI- scanner the stimuli were presented on a set of MRI- suited LCD- goggles (resolution 800 × 600; Resonance Technology, Northridge, CA, USA). If needed, the goggles were combined with corrective lenses to ensure corrected to normal vision. All participants wore headphones for communication with the experimenter and noise protection. Additionally, the participants underwent a masking and an emotional sentence task [43] in the scanner, which is not presented in this article. The total scan time was approximately 60 min. Thus, the study had a 2×3 quasi- experimental design with the within subject factor *word category* (TW, NW) and the between subject factor *group* (HDT, LDT, HC).

All stimuli had been selected previously in two pilot studies (unpublished data). In a first pilot study the valence of 69 words potentially relevant to tinnitus distress and 69 neutral words (matched for frequency of occurrence in German language, number of letters and syllables) was rated by 122 participants. Those participants were distributed evenly between three groups: high distressed tinnitus patients (TQ III and IV), low distressed tinnitus patients (TQ I and II) and healthy controls. The words were derived from the TQ, previous research, patient reports and interviews with medical and psychological tinnitus experts. From this study 28, emotionally relevant tinnitus words and 28 matched neutral words were selected. Emotional relevance was defined as a higher negative valence of the tinnitus- related words within the highly distressed group (maximized difference between the tinnitus and the neutral word) and also in comparison to the other two groups. In a second pilot study, 53 participants underwent an Emotional Stroop Task, 16 highly distressed tinnitus patients, 18 patients with low tinnitus distress and 19 healthy controls. Based on the results of the Stroop task, the six words with the biggest interference effect (response time to TW – response time to matched NW > 40 ms) within the HDT- group and with no interference effect in the LDT- and HC-group were selected for this study (see Table 2; Meinhardt-Renner and Kröner-Herwig unpublished).

**Table 2:** Stimuli: tinnitus and neutral words matched for word length and frequency of occurrence in the German language

Tinnitus Words	Neutral Words
Brummen	Kirsche
*to hum*	*cherry*
Nachdenken	Schubladen
*to think about sth.*	*drawers*
Nacht	Preis
*night*	*price*
Rauschen	Pflanzen
*static noise*	*to plant*
Schrill	Schrank
*shrill*	*cupboard*
Testbild	Weltmeer
*test pattern* ^a^	*ocean*

^a^The test pattern on German television screens was accompanied by a high pitched tone

### Assessment of psychosocial variables and audiological information

#### Tinnitus related distress

The TQ [38] is a self- report questionnaire consisting of 52 items. A total score of 0 to 30 corresponds to mild distress, a score between 31 and 46 matches moderate distress, a score of 47 to 59 corresponds to severe distress and a score of 60 and above is considered as very severe tinnitus distress [38]. The test- retest reliability of r_tt_ = 0.94 [44] can be considered as very good.

#### Determination of exclusion criteria

The German version of the Patient Health Questionnaire [45, 46] assesses diagnostic information about psychopathology and was used to exclude a major depressive syndrome and concurrent psychotropic medication. The Structured Tinnitus Interview (Strukturiertes Tinnitus Interview) [47] assesses detailed information about tinnitus and associated symptoms, such as hyperacusis, hearing loss and vertigo. It was used to exclude hyperacusis, hearing loss, acute tinnitus, non- continuous tinnitus and perception of tinnitus only in total silence.

An audiological evaluation was conducted to further exclude hearing loss and residual inhibition > one minute. Hearing level, minimal masking level, loudness discomfort level, residual inhibition, tinnitus pitch and loudness were assessed. With the exception of the hearing level and tinnitus loudness those features are not of any interest for the current study. The assessment was conducted in the clinical Department of Otorhinolaryngology of the University of Göttingen.

#### Sample characterization

Anxiety and depression scores were assessed with the German version of the HADS [39. 40]. Both subscales consist of seven items with a satisfactory internal consistency (anxiety subscale: α = 0.80, depression subscale: α = 0.81) and convergent validity (anxiety subscale: *r* = 0.65, depression subscale: *r* = 0.70). The scale has originally been developed for patients suffering from chronic medical conditions [39].

Hypersensitivity to sounds was assessed with the GÜF [41]. The questionnaire consists of 15 items and has a maximum score of 45; a score of 0–9 corresponds to mild hypersensitivity to sounds, 10 to 15 is considered as moderate, a score between 16 and 23 severe and 24 and above represents very severe hypersensitivity to sounds. Internal consistency for the subscales ranges between .77 and .82.

#### Behavioral data

To measure valence and arousal of the stimuli, the tinnitus and neutral words were rated on a computerized version of the Self- Assessment Manikin [48, 49]. The lower the values on the 9- point valence scale, the more negative a word is evaluated (1= very negative, 9= very positive). The higher the ratings on the 9- point arousal scale, the higher the arousal (1=not arousing, 9= very arousing). In order to test for an Emotional Stroop effect, response times of the color naming of the words were recorded during the MRI- scan.

#### Control variables

The vocabulary subtest (VT) of the Hamburg Wechsler Intelligence Test [47] was performed to control for differences in vocabulary, since novelty of words might act as a confounding variable [50].

### Image acquisition

MR imaging took place on a 3 T MRI- scanner (Siemens Magnetom TIM Trio, Siemens Healthcare, Erlangen, Germany). An 8- channel standard phased- array head coil was used (for one participant a 12- channel head coil was used due to head size). Firstly, an anatomical 3D T1- weighted dataset was attained (Turbo fast low angle shot (Turbo FLASH), echo time (TE): 3.26 ms, repetition time (TR): 2250 ms, inversion time: 900 ms, flip angle 12°) that covered the whole head at 1 × 1 × 1 mm^3^ isotropic resolution. T2*- weighted gradient- echo echo- planar imaging was used to acquire the functional datasets (TE: 36 ms, TR: 2000 ms, flip angle 90°, 22 slices of 4 mm thickness at an in- plane resolution of 2 × 2 mm^2^). Within one functional run 302 whole brain volumes were recorded.

### Procedure

Participants, who wanted to take part in the study, underwent a telephone- screening, which included questions regarding exclusion and inclusion criteria and the structured interview about tinnitus. Then, the participants were sent the following questionnaires: TQ, HADS- D, PHQ- D, GÜF and a specifically designed questionnaire to further assess MRI- specific exclusion criteria. In a next step the participants underwent the audiological examination (see above), which took part within one week before the MRI- examination. Before entering the MRI the participants underwent a pre- training to get familiar with the procedure. The Emotional Stroop pre- training consisted of four neutral words naming punctuation marks (Punkt (dot), Komma (comma), Fragezeichen (question mark), Klammer (bracket)) that appeared randomly in one of four different colors (red, blue, green, yellow). The participants were instructed to identify the colors via button press on a keyboard. The participants heard a feedback sound in case of a wrong or missing answer. After each block (16 trials, each word in each color) the instruction appeared again. The training program continued until the participant completed one run without mistakes to ensure all participants had successfully learned which buttons corresponded to which colors. After the pre- training the participants completed the EST inside the MRI- scanner without feedback. After the scanning procedure all participants evaluated the stimuli with the computerized version of the self- assessment Mannequin for arousal and valence and filled in the TQ for a second time. Additionally, the participants completed a vocabulary test, which was conducted via telephone on a later date, since they could be exhausted after the MRI- procedure.

### Statistical procedure

#### Behavioral data

The software STATISTICA (Version 10, Stat Soft. Inc., Tulsa, USA) was used to analyze the behavioral data. Regarding the reaction times in the Stroop Task and the ratings of valence and arousal three 3 x 2 repeated measures ANOVAs were performed with the between factor *group* (HDT, LDT, HC) and the within factor *word category* (TW, NW). If the sphericity assumption was violated, Greenhouse- Geisser corrections were performed. LSD- post- hoc- tests were performed and *p* was set at .05. As measure of dispersion the standard deviation of the mean was used throughout.

#### Functional imaging data

The fMRI data was analyzed with Brain Voyager QX Software version 2.0.8 (Brain Innovation, Maastricht, The Netherlands). Standard preprocessing was performed (3D motion correction, slice scan- time correction, temporal filtering (linear trend removal and high pass filtering) and spatial smoothing with a Gaussian kernel (full width at half maximum 8 × 8 × 8 mm^3^). On the basis of the general linear model, a random effects group analysis was performed. Only words to which participants responded correctly were used as predictors. Word stimuli with wrong or missing responses were included as confounding variables in the model. The effects of the 1750 ms presentation of the words were convolved with the canonical hemodynamic response function and analyses of planned contrasts were performed. Cluster level threshold estimation was used to correct for multiple comparisons [51, 52]. The uncorrected cluster threshold was set at *p* = .001 for within- group comparisons and correlational analyses (see below) and *p* = .005 for between- group comparisons. Monte Carlo simulations (1000 iterations) were performed on the basis of the estimated smoothness of the map and the number of activated voxels to determine the minimum cluster size which was required to yield a maximum error rate at the cluster level of *p* < .05. The Talairach Demon [53, 54] was used to identify activations by nearest coordinates. In accordance with the Four- Region Neurobiological Model [55–57] activations located in the cingulate gyrus were allocated to its subdivisions. Furthermore, the predictors for the contrast TW > NW were extracted and correlated with the individual TQ scores, HADS- depression and HADS- anxiety scores, the vocabulary test scores and the loudness of the tinnitus as assessed via tinnitus loudness matching (in dB HL). In the case of bilateral tinnitus, the louder tinnitus was included. Since there were differences between the groups in terms of vocabulary, anxiety and depression, those scores were included in a correlational analysis to check for potentially confounding effects. Tinnitus loudness was included to check for effects of salience. Recently, it has been suggested that the pain- matrix is not specific for nociceptive stimuli but reflects a salience detection system [58–60]. Therefore, in order to determine whether our effects are specific to the distress network we included a correlation with tinnitus loudness to explore activations within the salience network.

## Results

### Behavioral data

#### Reaction times, valence and arousal

**Fig. 2:**
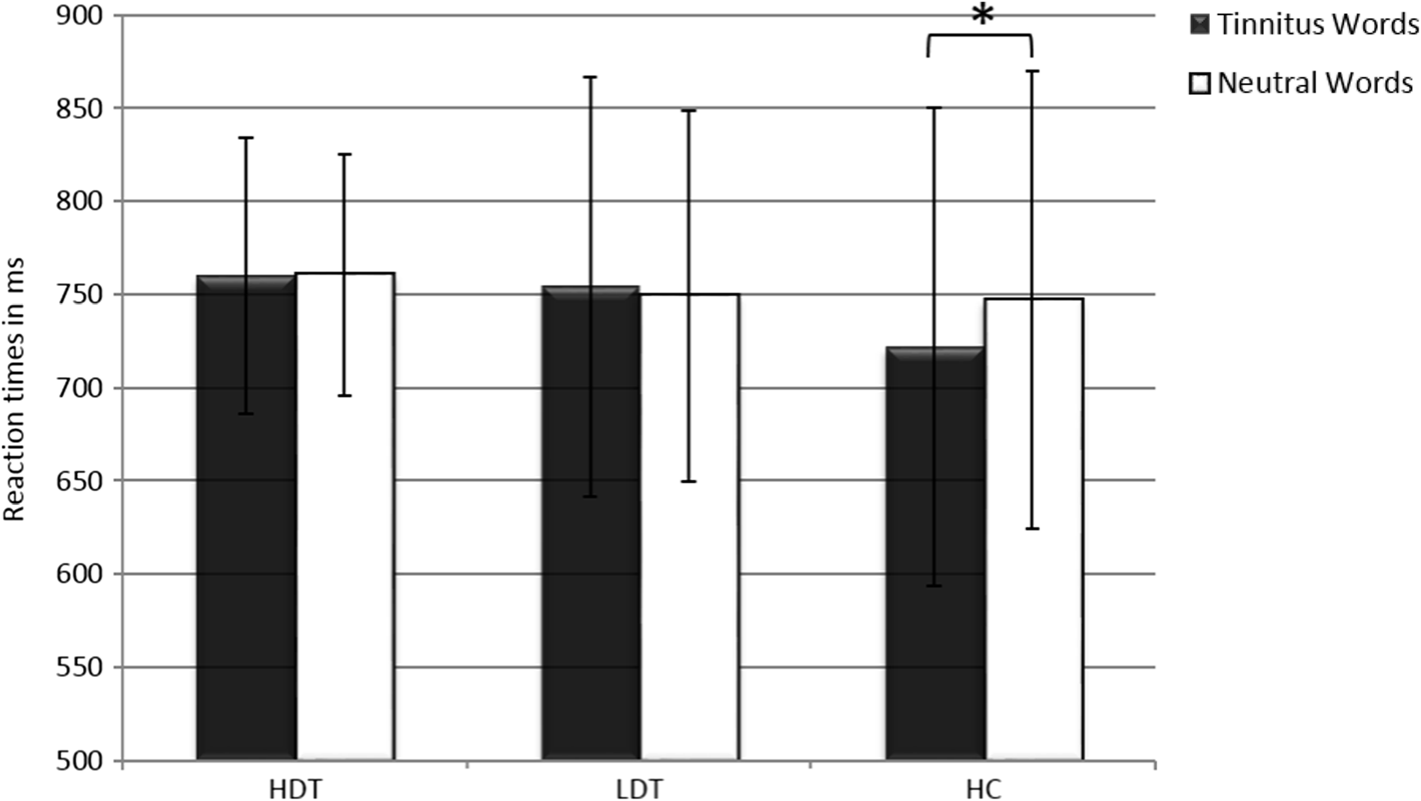
Reaction times in ms. HC= healthy controls, HDT= highly distressed tinnitus patients, LDT= low distressed tinnitus patients, ms= milliseconds, *= *p* < 0.05

It was expected that HDT would show slower reaction times to TW in comparison to NW. This difference should be greater for HDT in comparison to LDT and HC. A repeated measure ANOVA showed no main effect for group or word category, but a group x word category interaction; however LSD- post- hoc- tests revealed no differences within the HDT and LDT, but within the HC (see Fig. 2 and Table 3 for details).

**Table 3:** Behavioral data

							ANOVA		
	HDT		LDT		HC		Group	Word Cat.	G×W
	Mean	SD	Mean	SD	Mean	SD	*F* (2, 44)	*F* (1, 44)	*F* (2, 44)
Val TW	4.64	1.13	4.77	0.83	5.09	0.76	0.72	60.30	0.28
Val NW	5.83	1.29	5.93	0.49	6.05	0.95	*p*= 0.4941	*p*= 0.0000	*p*= 0.7555
Arou TW	3.35	1.70	2.88	1.33	3.16	1.73	0.30	12.44	0.28
Arou NW	2.72	1.67	2.38	1.42	2.78	1.85	*p*= 0.7399	*p*= 0.00099	*p*= 0.7544
RT TW	759.96	73.86	754.05	112.60	721.96	128.47	0.26	2.50	4.65
RT NW	760.40	64.60	748.97	99.79	746.82	122.71	*p*= 0.7688	*p*= 0.1212	*p*= 0.0146

*Arou* arousal, *F* F- value, *HC* healthy controls, *G* group, *HDT*= highly distressed tinnitus patients, *LDT* low distressed tinnitus patients, *NW* neutral words, *RT* reaction time, *SD* standard deviation, *TW* tinnitus words, *Val* valence, *Word Cat* word category

**Fig. 3:**
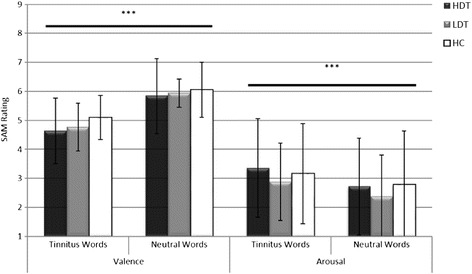
SAM- ratings of valence and arousal. Higher ratings correspond to a higher level of arousal and a more positive evaluation of the stimuli (valence). HC= healthy controls, HDT= highly distressed tinnitus patients, LDT= low distressed tinnitus patients, SAM= Self-Assessment-Manikin, ***= *p* < 0.001

Two repeated measures ANOVAs were conducted to assess differences with regard to valence and arousal ratings of the stimuli. According to valence and arousal we found a main effect for word category but no effect for group or a group x word category interaction. Thus, TW were rated more negative and arousing in comparison to NW (see Table 3 and Fig. 3 for details).

### FMRI data

#### Within group analysis

**Fig. 4:**
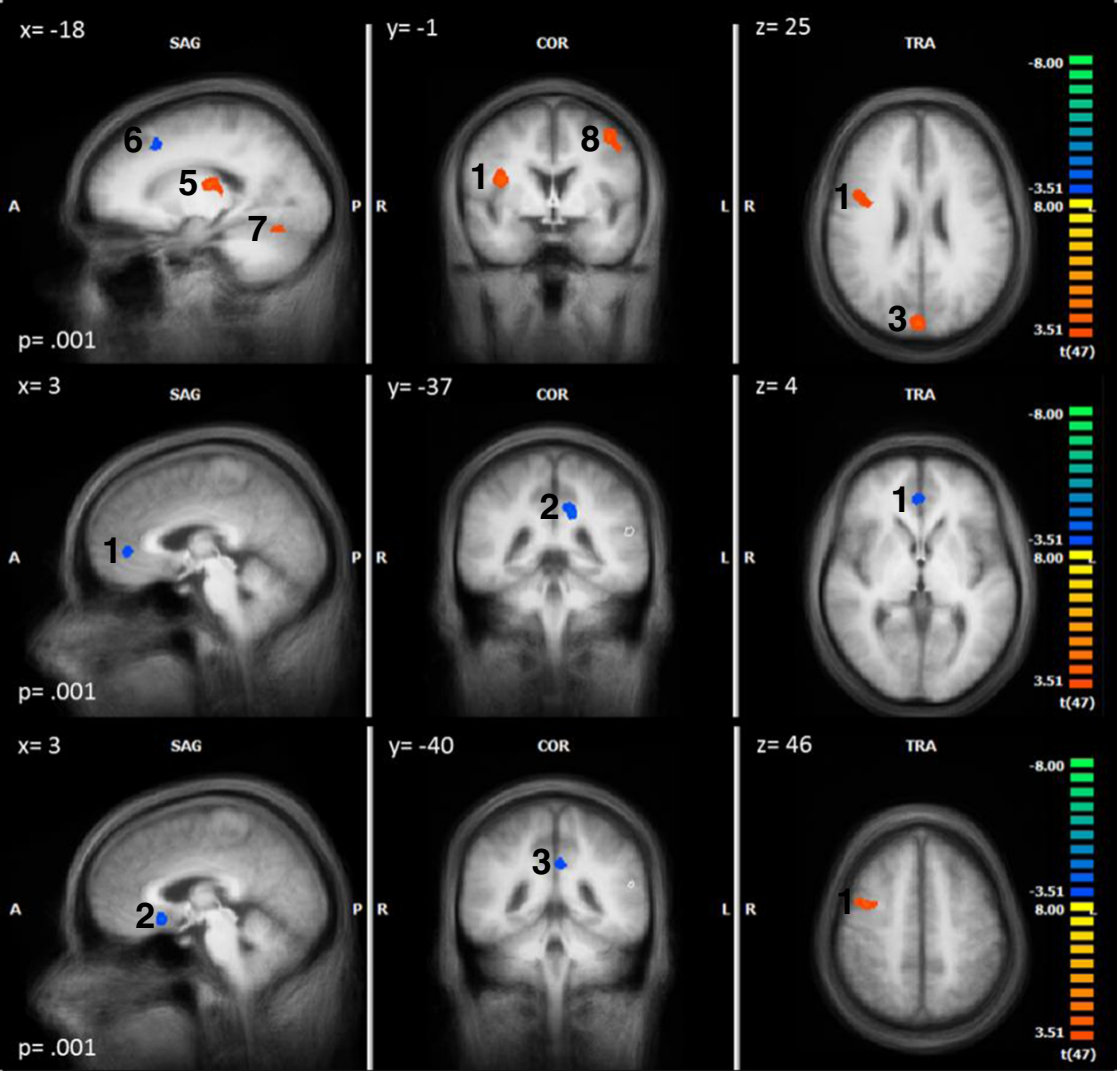
Within group results for HDT (*top*), LDT (*middle*) and HC (*bottom*) in the contrast TW - NW. The number next to each cluster corresponds to the cluster number in Table 4

Within each group the BOLD- response to TW was compared with the brain activity in reaction to NW. Within the HDT group we expected a higher BOLD- response in the precuneus, limbic and frontal areas, such as the cingulate gyrus, the parahippocampus, the insula, DLPFC and OFC. With regard to our hypothesis a higher BOLD- response to TW as compared to NW within the HDT group could be found in the right insula, right DLPFC and the right precuneus. The HC group showed higher activations to TW in right middle frontal regions, and higher activations to NW in the the left dorsal PCC and right subgenual ACC. LDT only showed higher activations to NW in the right perigenual ACC and left dorsal PCC (see Table 4 and Fig. 4 for details).

**Table 4:** Peak- voxels of the within- group results of the contrast TW - NW

Group	Region	BA	Peak Voxel			*t*	Cluster (mm^3^)
			x	y	z		
HDT	R Inferior Frontal Gyrus	09	45	8	22	4.20	1 (1755)
	R Insula	13	36	- 1	16	4.45	
	R Precentral Gyrus	06	30	- 10	52	3.86	2 (516)
	R Cuneus/Precuneus	07	6	- 73	34	3.64	3 (1665)
	L Cuneus	19	0	- 82	31	4.10	
	L Cuneus	18	- 3	- 94	11	3.87	
	L Thalamus		- 9	- 7	1	3.98	4 (279)
	L Thalamus		- 15	- 16	13	4.50	5 (985)
	L Superior Frontal Gyrus	08	- 15	24	46	- 4.24	6 (250)
	L Declive		- 18	- 64	- 17	3.83	7 (264)
	L Middle Frontal Gyrus	06	- 39	- 4	46	4.45	8 (1835)
	L Fusiform Gyrus		- 45	- 52	- 17	3.95	9 (268)
LDT	R pACC	32	3	41	4	- 3.86	1 (265)
	L dPCC	31	- 12	- 37	31	- 4.25	2 (368)
HC	R Middle Frontal Gyrus	09	51	11	34	4.09	1 (1245)
	R Middle Frontal Gyrus	06	39	2	46	4.10	
	R sACC	25	3	17	- 8	- 4.26	2 (254)
	L dPCC	31	- 3	- 40	31	- 3.97	3 (376)

*BA* Brodman area, *dPCC* dorsal posterior cingulate cortex, *HC* healthy controls, *HDT* highly distressed tinnitus patients, *L* left, *LDT* low distressed tinnitus patients, *pACC* perigenual anterior cingulate cortex, *R* right, *sACC* Subgenual anterior cingulate cortex, *t* t- value

#### Between group analysis

**Fig. 5:**
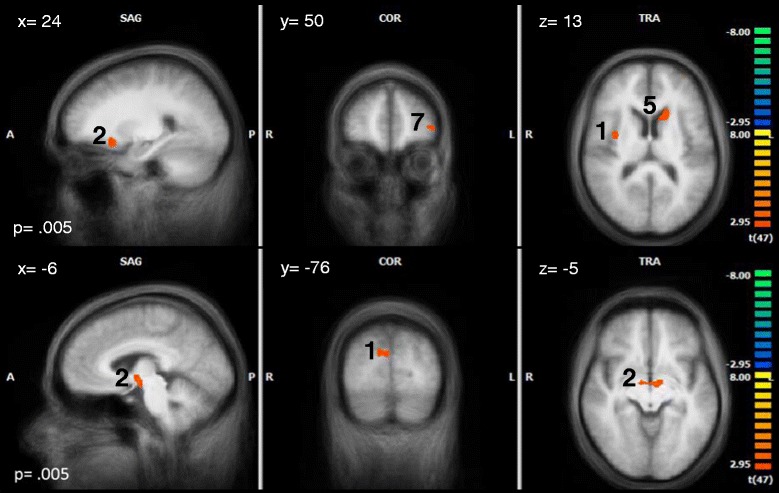
Between group results. The upper shows the contrast TW - NW in HDT vs. LDT (*top*), and for HDT vs. HC (*bottom*). The number next to each cluster corresponds to the cluster number in Table 5

**Fig. 6:**
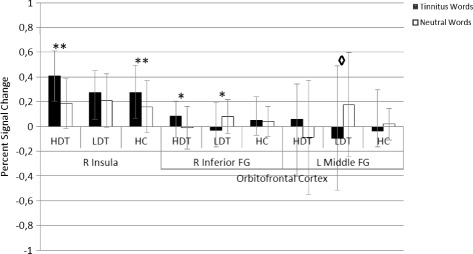
Percent signal change of the right insula and orbitofrontal cortex from the comparison HDT - LDT. FG= frontal gyrus, HC= healthy controls, HDT= highly distressed tinnitus patients, L= left, LDT= low distressed tinnitus patients, R= right, * *p*< .05, ** *p*< .01, ◊ p= .05

It was expected to find higher BOLD- responses in the hypothesized areas to TW in comparison to NW in HDT as compared to LDT and HC. We failed to find any differences in those regions when comparing HDT and HC, however we found a higher activation in the right insula and the OFC in the HDT group as compared to the LDT group (see Table 5 and Fig. 5 for details). Fig. 6 shows the percent signal change of the right insula and the orbitofrontal cortex.

**Table 5:** Peak- voxels of the between- group results

TW - NW	Region	BA	Peak Voxel			*t*	Cluster (mm^3^)
			x	y	z		
HDT vs. LDT	R Insula	13	33	- 1	13	3.81	1 (215)
	R Inferior Frontal Gyrus	47	24	17	- 8	3.40	2 (439)
	R Cuneus	18	3	- 79	25	3.64	3 (1186)
	L Hypothalamus		- 9	- 4	- 2	4.80	4 (2598)
	L Lentiform Nucleus		- 24	- 10	- 5	3.72	
	L Caudate		- 15	17	13	3.93	5 (385)
	L Postcentral Gyrus	03	- 24	- 30	61	3.90	6 (117)
	L Middle Frontal Gyrus	10	- 39	50	7	3.16	7 (199)
HDT vs. HC	R Hypothalamus		9	- 7	- 8	3.41	1 (877)
	L Hypothalamus		- 6	- 7	- 5	4.16	
	R Cuneus	18	12	- 76	25	3.27	2 (208)

*BA* Brodman area, *HC* healthy controls, *HDT* highly distressed tinnitus patients, *L* left, *LDT* low distressed tinnitus patients, *R* right, *t* t- value

#### Correlational analysis

**Fig. 7:**
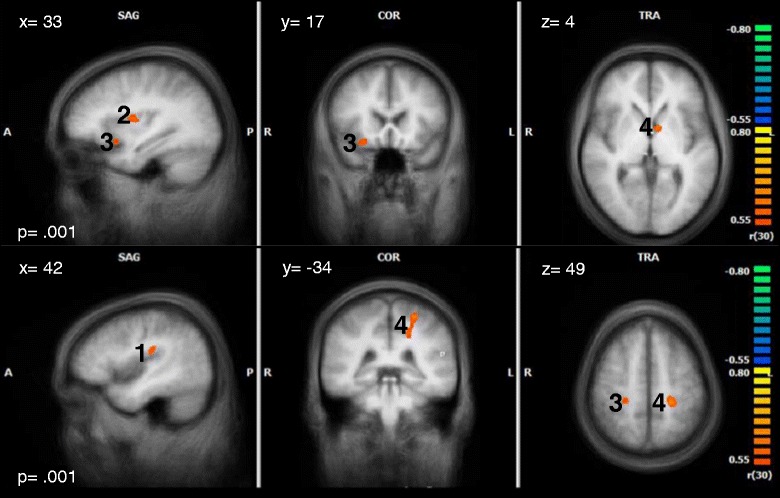
Correlations with tinnitus distress and depression

We further correlated the beta weights for the contrast TW > NW with tinnitus distress within the tinnitus group (HDT and LDT). Furthermore, correlations were computed with tinnitus loudness and all variables that differed between HDT and LDT. Correlations with tinnitus distress were found for the right insula and the right inferior frontal gyrus as part of the OFC. Depression correlated positively with activity in the right insula and the left dorsal PCC (see Table 6 and Fig. 7 for details). No other correlations were found.

**Table 6:** Peak- voxels of the correlations between the contrast TW - NW and TQ- scores, depression scores, anxiety scores, vocabulary test scores, GÜF-scores and maximum tinnitus loudness (in dB)

TW - NW	Region	BA	Peak Voxel			*r* (*p*= .001)	Cluster (mm^3^)
			x	y	z		
TQ	R Transverse Temporal Gyrus	41	45	- 22	13	0.60	1 (117)
	R Insula	13	33	- 1	13	0.62	2 (217)
	R Inferior Frontal Gyrus	47	24	17	- 11	0.62	3 (269)
	L Caudate		- 6	2	4	0.62	4 (248)
HADS- D	R Insula	13	42	- 22	22	0.61	1 (110)
	R Postcentral Gyrus	03	24	- 28	49	0.60	2 (123)
	L Thalamus		- 12	- 22	13	0.62	3 (129)
	L dPCC	31	- 18	- 34	37	0.60	4 (1503)
	L Postcentral Gyrus	03	- 24	- 31	52	0.70	4
HADS- A	No correlation						
VT	No correlation						
GÜF	No correlation						
Loudness	No correlation						

*BA* Brodman area, *dPCC* dorsal posterior cingulate cortex, *GÜF* Geräuschüberempfindlichkeitsfragebogen (Questionnaire on Hypersensitivity to sound), *HADS* Hospital Anxiety (A) and Depression (D) Scale, *L* left, *NW* neutral words, *r* correlation coefficient, *R* right, *TQ* Tinnitus Questionnaire, *TW* tinnitus-related words, *VT* vocabulary test

The figure shows the correlation between the contrast TW - NW and the TQ- scores (top), and the correlation between TW - NW and the HADS depression scores (bottom) (only tinnitus patients were included). The number next to each cluster corresponds to the cluster number in table 6.

## Discussion

The aim of the study was to examine possible effects of selective attention and the emotional processing of tinnitus- related words and their relation to tinnitus distress. Therefore an EST was conducted and the neural activity elicited by TW was compared to the neural response to NW within the HDT group and in comparison to LDT and HC. Furthermore the BOLD- response to TW was correlated with tinnitus distress, tinnitus loudness, vocabulary, depression, anxiety and hypersensitivity to sound. It was expected to find longer reaction times between TW and NW within HDT and in comparison to LDT and HC. Furthermore HDT should evaluate TW as more arousing and negative than NW and compared to the other two groups. However, we did not find any hypothesized effects of response times, nor did we find differences between HDT and the other two groups with regard to valence and arousal. All groups rated TW as more negative and arousing as compared to NW. On a neural level though, the HDT group showed a higher activation in the right insula and bilaterally in the OFC as compared to LDT. Furthermore, tinnitus distress correlated positively with the BOLD- response in the right insula and the right inferior frontal gyrus as part of the OFC. Activity in the right insula and the left dorsal PCC correlated positively with depression. Contradictory to our predictions we did not find differences between HDT and HC in any of the hypothesized regions. Thus, on a neural level our hypotheses have been partially supported.

### The lack of an emotional stroop effect in HDT

Possible explanations for the lack of an Emotional Stroop effect are the response modality, type of stimuli and the infeasibility of the visual modality to examine effects of selective attention in tinnitus patients. It has been shown that a response via button-press, as in the current study, leads to smaller interference effects as compared to a vocal response in the original Stroop task [61]. However, since tinnitus is a heterogeneous symptom with great variations in variables such as tinnitus location and tinnitus pitch [62] standardized stimuli might not be the best choice. Idiosyncratic word stimuli which are more relevant to the individual emotional concerns (e.g. worries about the tinnitus) of each tinnitus patient could lead to better results. Studies using idiosyncratic word stimuli found Stroop effects in various areas such as posttraumatic stress disorder [63], obsessive- compulsive disorder [64] and healthy subjects [65]. However, no Emotional Stroop effect could be found in chronic pain patients [66], who share common features with tinnitus patients [23, 24, 67], though idiographic stimuli had been used.

Thus, paradigms that examine auditory selective attention might be more suitable to find differences not only on a neural, but also on a behavioral level. For example, in a dichotic listening task it has been shown that alcohol- dependent inpatients show more shadowing errors in comparison to social drinkers when concern- related words were presented in the irrelevant channel as compared to neutral words [68]. In an associative learning procedure [69], 42 different click- like tones were conditioned with positive, negative or neutral sounds from the International Affective Digitized Sounds system [70]. Magnetoencephalography showed an intensified processing of tones associated with emotional sounds (negative or positive) as compared to neutral sounds in frontal, parietal and auditory sensory areas. Thus, dichotic listening tasks that use tinnitus- related words or affective conditioning paradigms might be another possibility to examine effects of selective attention in tinnitus patients. However, a third possibility, which we cannot rule out in this study, might be a lack of power, since the only study, which found a facilitation effect in tinnitus- patients for tinnitus- related words consisted of 104 participants.

Though the current study did not find an interference effect, the fMRI- results can still be interpreted as neural correlates of tinnitus-related distress. An example from EEG-experiments even shows that neural responses could be more sensitive than reaction times [71, 72]. The N400 differentiated well between two conditions (semantically related vs. unrelated) in a letter-search priming paradigm in the absence of a reaction time effect, indicating a semantic context effect [72]. Thus, the authors believe that the results indicate the emotional processing of tinnitus- related words; however the emotional salience of those words obviously was not strong enough to interfere with the task. Thus future studies should use individual tinnitus words to ensure a high personal relevance of the stimuli as discussed above.

### Differences between the groups

The amount of personal relevance of the stimuli could also explain the lack of hypothesized differences between HDT and HC, since the TW could not only be interpreted as tinnitus associated stimuli, but also by HC as generally negative characteristics (e.g. a shrill voice). This view is supported by earlier results, in which HDT showed among others a higher activation in the right insula to tinnitus- related sentences as compared to neutral sentences within their group and in comparison to HC [43]. The sentences provided a clear tinnitus context (e.g. *I will never get rid of the tinnitus*). Furthermore, the personal relevance of the sentences was rated and HDT evaluated tinnitus- related sentences as being more personally relevant in comparison to generally negative sentences, additionally they rated tinnitus sentences higher as compared to HC. HC however evaluated neutral sentences as more personally relevant than tinnitus- related and generally negative sentences. Thus, it might indeed be beneficial for future studies to include tinnitus- related words which are personally relevant to tinnitus patients but not for HC.

However, a number of resting- state studies, as mentioned above, found those differences. Thus, this finding might also be due to the methodology of a task- driven approach. LDT might have actively avoided the tinnitus words. This view is supported by the percent signal change in the OFC. While HDT tend to show higher activations to TW as compared to NW, this pattern seems to be reversed in low distressed patients. It has been shown before that reappraisal, as a strategy of emotional regulation, could lower the activation within the orbitofrontal cortex [73] and the insula [74]. Thus, an additional down- regulation of negative emotions in the low distressed group could explain the differences between HDT and LDT.

### Tinnitus distress and depression

Activity in the right insula correlated with both; tinnitus distress and depression. Recently, using partial correlations, it has been found that tinnitus distress correlated exclusively with current density distribution in alpha 2, beta 1 and beta 2 activity of the *right* OFC and frontopolar cortex and beta 2 activation of the ACC. Depression scores however correlated with alpha 2 activity in the *left* OFC and frontopolar cortex [75]. This lateralization effect could however not be confirmed in this study. A recently conducted meta- analysis [76] showed that depressed individuals show a higher activation to negative stimuli in the amygdala, insula and dorsal ACC and a lower activation in the dorsal striatum and DLPFC as compared to healthy controls. Our results suggest the insula to play a major role in the distress network; however this activation seems not to be specific for distress, but also for depression. It has been shown before that tinnitus distress and depression are associated with each other in a 2- year longitudinal study on 6215 people from the Swedish working population [77]. Furthermore, the HDT and the LDT group differed not only with regard to tinnitus distress, but also in depression, anxiety, vocabulary and hypersensitivity to sounds. However, aside from depression, none of these variables correlated with the BOLD- response. Thus, it may be that tinnitus distress and depression activate overlapping brain networks; an idea which has been proposed earlier [78] and which is conform with the assumption of an unspecific distress network [79].

### Multiple overlapping networks

Since we tested HDT and LDT, the distress network, which according to De Ridder et al. [28] includes the anterior insula, amygdala and ACC, should be more active in HDT. Indeed we found the right insula to be more active within HDT and in comparison to LDT. However, the anterior insula is supposed to be part of the distress *and* the salience network [35]. According to a meta-analysis about the functional differentiation of the insula [80] the dorsal part of the anterior insula is a highly integrative region of multiple processes, such as emotional-cognitive processing and interoception. The activation of the insula in the current study seems to be located in the central part of the insula, which is associated with interoception [80]. Interoception on the other hand is closely linked to the perception of emotions [81–83]. Thus, in an experiment in which the heartrate-feedback was manipulated participants evaluated neutral faces as being more emotional, if they received a false feedback of an accelerated heartbeat. Higher activity within the right anterior insula was associated with higher emotionality ratings during false feedback [81].

In the field of pain research it has been suggested that the so- called pain- matrix does not reflect activations specific to nociceptive stimulation but rather the behavioral significance of a stimulus regardless of its modality [58–60]. In the field of tinnitus research it might also be important to differentiate between the salience of tinnitus, which could be reflected by its loudness and tinnitus distress. We, however, found a correlation between the BOLD- response in the right insula and tinnitus distress, but not with tinnitus loudness. Thus, the activation of the right insula in our sample might indeed reflect tinnitus distress rather than its salience.

### Limitations

There are some limitations to the current study. A problem which is directly related to tinnitus research might be the scanner noise [84, 85]. The scanner noise could mask the participant’s tinnitus [84] and even have differential effects on non- auditory brain areas subject to the cognitive demand of the task [86]. Since our study used verbal material it was not important whether the tinnitus was masked by the scanner noise. Furthermore we did not vary the cognitive demand of tasks between the groups, since both groups saw exactly the same stimuli and were given the same instructions. In addition, we controlled for hearing loss. Thus, differential effects of scanner noise are unlikely. Another issue could be the level of distress in the HDT group, since most of the participants in this group had only moderate levels of tinnitus distress. However, moderately distressed tinnitus patients often take part in studies on the effect of cognitive- behavioral therapies that aim to reduce tinnitus- related distress [87–89]. This indicates that moderately distressed tinnitus patients differ from LDT in their help seeking behavior.

### Implications for future studies

For future studies of the neural correlates of tinnitus distress, a combination of resting- state and task- driven fMRI approaches might be useful to make the results more comparable. The resting- state could be assessed via EEG and fMRI. Idiosyncratic word stimuli relevant to tinnitus- related concerns should be used as stimulus material in a sample of HDT who should be scanned twice; before and after a cognitive behavioral intervention. Cognitive- behavioral interventions would be the method of choice, since they have reliably shown to be effective in reducing tinnitus- related distress [90]. A repeated measures design pre and post therapy would have the advantage of investigating changes in the distress network and help to identify cortical hubs in tinnitus distress. Furthermore it would help to compare resting- state analysis with a task- driven approach.

## Conclusion

Tinnitus-related words seem to activate the distress network in HDT. The roles of the insula and the OFC in the distress network have been confirmed by a task-driven fMRI-approach. Additionally, LDT seem to actively avoid tinnitus-related stimuli. The distress network and depression network seem to partially overlap in their activation of the right insula. Prospective studies are needed to further explore the distress network in chronic tinnitus.

## Abbreviations

ACC, anterior cingulate cortex; Arou, arousal; BA, Brodman Area; BOLD, blood oxygen level dependent; dB, decibel; df, degrees of freedom; DLPFC, dorsolateral prefrontal cortex; dPCC, dorsal posterior cingulate cortex; EEG, electroencephalogram; EST, emotional stroop task; F, F- value; fMRI, functional magnetic resonance imaging; GÜF, geräuschüberempfindlichkeitsfragebogen (Questionnaire on hypersensitivity to sound); HADS A, hospital anxiety and depression inventory, anxiety subscale; HADS D, hospital anxiety and depression inventory, depression subscale; HADS, hospital anxiety and depression Inventory; HC, healthy controls; HDT, highly distressed tinnitus patients; HL, hearing level; L, left; LDT, low distressed tinnitus patients; ms, milliseconds; NW, neutral words; OFC, orbitofrontal cortex; pACC, perigenual anterior cingulate cortex; PCC, posterior cingulate cortex; PFC, prefrontal cortex; R, right; RT, reaction time; sACC, subgenual anterior cingulate cortex; SD, standard deviation; t, t- value; TE, echo time; TQ, tinnitus questionnaire; TR, repetition time; TW, tinnitus words; Val, valence; VT, vocabulary test; Word Cat., word category

## References

[1] Møller A, Møller A, Langguth B, De Ridder D, Kleinjung T (2011). Introduction. Textbook of Tinnitus.

[2] Palmer KT, Griffin MJ, Syddall HE, Davis A, Pannett B, Coggon D (2002). Occupational exposure to noise and the attributable burden of hearing difficulties in Great Britain. Occup Environ Med.

[3] Fabijanska A, Rogowski M, Bartnik G, Skarzynski H, Hazell J (2007). Epidemiology of tinnitus and hyperacusis in Poland. Proceedings of the Sixth International Tinnitus Seminar.

[4] Axelsson A, Ringdahl A (1989). Tinnitus- a study of its prevalence and characteristics. Br J Audiol.

[5] Henry JA, Meikle MB (2000). Psychoacoustic measures of tinnitus. J Am Acad Audiol.

[6] Hiller W, Goebel G (2007). When tinnitus loudness and annoyance are discrepant, audiological characteristics and psychological profile. Audiol Neurootol.

[7] Jastreboff PJ, Gray WC, Gold SL (1996). Neurophysiological approach to tinnitus patients. Am J Otol.

[8] Hallam RS, Hazell JWP (1987). Psychological approaches to the evaluation and management of tinnitus distress. Tinnitus.

[9] Mertin M, Kröner- Herwig B, Kröner- Herwig B (1997). Tinnitus aus psychologischer Sicht. Psychologische Behandlung des chronischen Tinnitus.

[10] Cuny C, Norena A, Massioui F, Chéry- Croze S (2004). Reduced attention shift in response to auditory changes in subjects with tinnitus. Audiol Neurootol.

[11] Rief W, Sander E, Günther M, Nanke A (2004). Aufmerksamkeitslenkung bei Tinnitus: Eine experimentelle psychophysiologische Untersuchung. Z Klin Psychol Psychother.

[12] Delb W, Strauss DJ, Low YF, Seidler H, Rheinschmitt A, Wobrock T, D’Amelio R (2008). Alterations in event related potentials, ERP associated with tinnitus distress and attention. Appl Psychophysiol Biofeedback.

[13] Andersson G, Westin V (2008). Understanding tinnitus distress: Introducing the concepts of moderators and mediators. Int J Audiol.

[14] Cima RFF, Crombez G, Vlaeyen JWS (2011). Catastrophizing and fear of tinnitus predict quality of life in patients with chronic tinnitus. Ear Hear.

[15] Andersson G, Jüris L, Classon E, Frederikson M, Furmark T (2006). Consequences of suppressing thoughts about tinnitus and the effects of cognitive distraction on brain activity in tinnitus patients. Audiol Neurootol.

[16] Andersson G, Raghad B, Johansson L, Kaldo V, Carlbring P (2005). Stroop facilitation in tinnitus patients: An experiment conducted via the world wide web. Cyberpsychol Behav.

[17] Malhi GS, Lagopoulos J, Sachdev PS, Ivanovski B, Shnier R (2005). An emotional Stroop functional MRI study of euthymic bipolar disorder. Bipolar Disord.

[18] Posner J, Maia TV, Fair D, Peterson BS, Sonuga-Barke EJ, Nagel BJ (2011). The attenuation of dysfunctional emotional processing with stimulant medication: an fMRI study of adolescents with ADHD. Psychiatry Res Neuroimaging.

[19] Mohanty A, Engels AS, Herrington JD, Heller W, Ringo Ho MH, Banich MT (2007). Differential engagement of anterior cingulate cortex subdivisions for cognitive and emotional function. Psychophysiology.

[20] Williams JMG, Mathews A, Macleod C (1996). The Emotional Stroop task and psychopathology. Psychol Bull.

[21] Roelofs J, Peters ML, Zeegers MPA, Vlaeyen JWS (2002). The modified Stroop paradigm as a measure of selective attention towards pain- related stimuli among chronic pain patients: a meta- analysis. Eur J Pain.

[22] Møller A (2007). The role of neural plasticity in tinnitus. Prog Brain Res.

[23] Folmer RL, Griest SE, Martin WH (2001). Chronic tinnitus as phantom auditory pain. Otolaryngol Head Neck Surg.

[24] Tonndorf J (1987). The analogy between tinnitus and pain: A suggestion for a physiological basis of chronic tinnitus. Hear Res.

[25] Cisler JM, Wolitzky-Taylor KB, Adams TG, Babson KA, Badour CL, Willems JL (2011). The emotional Stroop task and posttraumatic stress disorder: a meta-analysis. Clin Psychol Rev.

[26] Andersson G, Eriksson J, Lundh LG (2000). Tinnitus and cognitive interference: a Stroop paradigm study. JSLHR.

[27] Schlee W, Lorenz I, Hartmann T, Müller N, Schulz H, Weisz N, Møller A, Langguth B, De Ridder D, Kleinjung T (2011). A global brain model of tinnitus. Textbook of Tinnitus.

[28] De Ridder D, Vanneste S, Congedo M (2011). The distressed brain: A group blind source separation analysis on tinnitus. PLoS One.

[29] Vanneste S, Plazier M, van der Loo E, van de Heyning P, Congedo M, De Ridder D (2010). The neural correlates of tinnitus- related distress. Neuroimage.

[30] Hallam RS (1996). Manual of the Tinnitus Questionnaire, TQ.

[31] Maudoux A, Lefebvre P, Cabay JE, Demertzi A, Vanhaudenhuyse A, Laureys S, Soddu A (2012). Auditory resting- state network connectivity in tinnitus: a functional MRI study. PLoS One.

[32] Burton H, Wineland A, Bhattacharya M, Nicklaus J, Garcia KS, Piccirillo JF. Altered networks in bothersome tinnitus: a functional connectivity study. BMC Neuroscience. 2012. doi:10.1186/1471-2202-13-3. 10.1186/1471-2202-13-3 PMC3282646 22217183

[33] Wineland AM, Burton H, Piccirilo J (2012). Functional connectivity networks in nonbothersome tinnitus. Otolaryngol Head Neck Surg.

[34] Schlee W, Mueller N, Hartmann T, Keil J, Lorenz I, Weisz N. Mapping cortical hubs in tinnitus. BMC Biol. 2009. doi:10.1186/1741-7007-7-80. 10.1186/1741-7007-7-80 PMC2787501 19930625

[35] De Ridder D, Elgoyhen AB, Romo R, Langguth B (2011). Phantom percepts: Tinnitus and pain as persisting aversive memory networks. Proc Natl Acad Sci U S A.

[36] Vanneste S, De Ridder D (2012). The auditory and non- auditory brain areas involved in tinnitus. An emergent property of multiple parallel overlapping subnetworks. Front Syst Neurosci..

[37] Schwerhörigkeit SH (1996). Ursachen, Diagnostik, Therapie, Hörgeräteversorgung.

[38] Goebel G, Hiller W (1998). Tinnitus- Fragebogen, TF. Ein Instrument zur Erfassung von Belastung und Schweregrad bei Tinnitus. Handanweisung.

[39] Herrmann C, Buss U, Snaith RP (1995). HADS- D Hospital Anxiety and Depression Scale - Deutsche Version.

[40] Snaith RP, Zigmond AS (1994). HADS: Hospital Anxiety and Depression Scale.

[41] Nelting M, Finlayson NK (2004). GÜF Geräuschüberempfindlichkeits- Fragebogen.

[42] Tewes U (1991). HAWIE- R Hamburg- Wechsler Intelligenztest für Erwachsene Revision.

[43] Golm D, Schmidt- Samoa C, Dechent P, Kröner- Herwig B (2013). Neural correlates of tinnitus related distress: An fMRI study. Hear Res.

[44] Hiller W, Goebel G, Rief W (1994). Reliability of self- rated tinnitus distress and association with psychological symptom patterns. Br J Clin Psychol.

[45] Gräfe K, Zipfel S, Herzog W, Löwe B (2004). Screening psychischer Störungen mit dem “Gesundheitsfragebogen für Patienten, PHQ- D” Ergebnisse der deutschen Validierungsstudie. Diagnostica.

[46] Spitzer RL, Kroenke K, Williams JBW (1999). Validation and utility of a self- report version of PRIME- MD: The PHQ primary care study. JAMA.

[47] Goebel G, Hiller W (2001). Verhaltensmedizinische Tinnitus- Diagnostik. Eine praktische Anleitung zur Erfassung medizinischer und psychologischer Merkmale mittels des Strukturierten Tinnitus Interviews, STI.

[48] Bradley MM, Lang PJ (1994). Measuring emotion– the self- assessment mannequin and the semantic differential. J Behav Ther Exp.

[49] Barke A, Stahl J, Kröner- Herwig B (2011). Identifying a subset of fear- evoking pictures from the IAPS on the basis of dimensional and categorical ratings for a German sample. J Behav Ther Exp Psychiatry.

[50] Saykin AJ, Johnson SC, Flashman LA, McAllister TW, Sparling M, Dracey TM, Moritz CH, Guerin DJ, Weaver J, Mamourian A (1999). Functional differentiation of medial temporal and frontal regions involved in processing novel and familiar words: an fMRI study. Brain.

[51] Forman SD, Cohen JD, Fitzgerald M, Eddy WF, Mintun MA, Noll DC (1995). Improved assessment of significant activation in functional magnetic resonance imaging, fMRI: use of a cluster- size threshold. Magn Reson Med.

[52] Goebel R, Esposito F, Formisano E (2006). Analysis of functional image analysis contest, FIAC data with Brainvoyager QX: from single- subject to cortically aligned group general linear model analysis and self- organizing group independent component analysis. Hum Brain Mapp.

[53] Lancaster JL, Rainey LH, Summerlin JL, Freitas CS, Fox PT, Evans AC, Toga AW, Mazziotta JC (1997). Automated labeling of the human brain: a preliminary report on the development and evaluation of a forward transform method. Hum Brain Mapp.

[54] Lancaster JL, Woldorff MG, Parsons LM, Liotti M, Freitas CS, Rainey L, Kochunov PV, Nickerson D, Mikiten SA, Fox PT (2000). Automated Talairach atlas labels for functional brain mapping. Hum Brain Mapp.

[55] Vogt BA, Berger GR, Derbyshire SWG (2003). Structural and functional dichotomy of human midcingulate cortex. Eur J Neurosci.

[56] Vogt BA, Hof PR, Vogt LJ, Paxinos G, Mai JK (2004). Cingulate Gyrus. The human nervous system.

[57] Vogt BA (2005). Pain and emotion interactions in subregions of the cingulate gyrus. Nat Rev Neurosci.

[58] Iannetti GD, Mouraux A (2010). From the neuromatrix to the pain matrix, and back. Exp Brain Res.

[59] Legrain V, Iannetti GD, Plaghki L, Mouraux A (2011). The pain matrix reloaded: A salience detection system for the body. Prog Neurobiol.

[60] Mouraux A, Diukova A, Lee MC, Wise RG, Iannetti GD (2011). A multisensory investigation of the functional significance of the “pain matrix”. Neuroimage.

[61] MacLeod CM (1991). Half a century research on the Stroop effect: an integrative review. Psychol Bull.

[62] Møller AR, Møller AR, Langguth B, De Ridder D, Kleinjung T (2011). Different forms of tinnitus. Textbook of tinnitus.

[63] Kaspi SP, McNally RJ, Amir N (1995). Cognitive processing of emotional information in posttraumatic stress disorder. Cognit Ther Res.

[64] Amir N, Najmi S, Morrison AS (2009). Attenuation of attention bias in obsessive- compulsive disorder. Behav Res Ther.

[65] Riemann BC, McNally RJ (1995). Cognitive processing of personally relevant information. Cognit Emot.

[66] Roelofs J, Crombez G, Peters ML, Verschuere B, Vlaeyen JWS (2005). An examination of word relevance in a modified stroop task in patients with chronic low back pain. Percept Mot Skills.

[67] Møller AR (2007). Tinnitus and pain. Prog Brain Res.

[68] Stetter F, Ackermann K, Scherer E, Schmid H, Straube ER, Mann K (1994). Distraction resulting from disease related words in alcohol- dependent inpatients: a controlled dichotic listening study. Eur Arch Psychiatry Neurol Sci.

[69] Bröckelmann AK, Steinberg C, Elling L, Zwangzger P, Pantev C, Junghöfer M (2011). Emotion- associated tones attract enhanced attention at early auditory processing: magnetoencephalographic correlates. J Neurosci.

[70] Bradley MM, Lang PJ (2000). Affective reactions to acoustic stimuli. Psychophysiology.

[71] Heil M, Rolke B (2004). Unattended distractor-induced priming in a visual selective attention task: N400 effects in the absence of RT effects. J Psychophysiol.

[72] Heil M, Rolke B, Pecchinenda A (2004). Automatic semantic activation is no myth: semantic context effects on the N400 in the letter-search task in the absence of response time effects. Psychol Sci.

[73] Ochsner KN, Bunge SA, Gross JJ, Gabrieli JDE (2002). Rethinking feelings: an fMRI study of the cognitive regulation of emotion. J Cogn Neurosci.

[74] Goldin PR, McRae K, Ramel W, Gross JJ (2008). The neural bases of emotion regulation: reappraisal and suppression of negative emotion. Biol Psychiatry.

[75] Joos K, Vanneste S, De Ridder D (2012). Disentangling depression and distress networks in the tinnitus brain. PloS One.

[76] Hamilton JP, Etkin AE, Furman DJ, Lemus MG, Johnson RF, Gotlib IH (2012). Functional neuroimaging of major depressive disorder: A meta-analysis and new integration of baseline activation and neural response data. Am J Psychiat.

[77] Hébert S, Canlon B, Hasson D, Magnusson Hanson LL, Westerlund H, Theorell T (2012). Tinnitus severity is reduced with reduction of depressive mood – a prospective population study in Sweden. PLoS One.

[78] Langguth B, Landgrebe M, Møller A, Langguth B, De Ridder D, Kleinjung T (2011). Textbook of Tinnitus.

[79] De Ridder D, Møller A, Langguth B, DeRidder D, Kleinjung T (2011). A Heuristic pathophysiological model of tinnitus. Textbook of Tinnitus.

[80] Kurth F, Zilles K, Fox PT, Laird AR, Eickhoff SB (2010). A link between the systems: Functional differentiation and integration within the human insula revealed by meta-analysis. Brain Struct Funct.

[81] Gray MA, Harrison NA, Wiens S, Crichley HD (2007). Modulation of emotional appraisal by false physiological feedback during fMRI. PLoS One.

[82] Singer T, Critchley HD, Preuschoff K (2009). A common role of insula in feelings, empathy and uncertainty. Trends Cogn Sci.

[83] Critchley HD (2005). Neural Mechanisms of autonomic, affective, and cognitive integration. J Comp Neurol.

[84] Danesh AA, Kinouchi Y, Wener DL, Pandya A, Palade V, Howlett RJ, Jain LC (2003). Functional imaging of tinnitus: seeing of the unseeable. Knowledge- based intelligent information and engeneering systems, lecture notes in computer science.

[85] Lockwood AH, Burkhard RF, Salvi RJ, Snow JB (2004). Imaging tinnitus. Tinnitus: theory and management.

[86] Tomasi D, Caparelli EC, Chang L, Ernst T (2005). fMRI- acoustic noise alters brain activation during working memory tasks. Neuroimage.

[87] Kröner- Herwig B, Frenzel A, Fritsche G, Schilkowsky G, Esser G (2003). The management of chronic tinnitus. Comparison of an outpatient cognitive behavioral group training to minimal contact interventions. J Psychosom Res.

[88] Zachriat C, Kröner- Herwig B (2004). Treating chronic tinnitus: comparison of cognitive- behavioural and habituation- based treatments. Cogn Behav Ther.

[89] Rief W, Weise C, Kley N, Martin A (2005). Psychophysiological treatment of chronic tinnitus: a randomized clinical trial. Psychosom Med.

[90] Hesser H, Weise C, Westin VZ, Andersson G (2011). A systematic review and meta- analysis of randomized controlled trials of cognitive- behavioral therapy for tinnitus distress. Clin Psychol Rev.

